# General Trends in Core–Shell Preferences for Bimetallic Nanoparticles

**DOI:** 10.1021/acsnano.1c01500

**Published:** 2021-04-23

**Authors:** Namsoon Eom, Maria E Messing, Jonas Johansson, Knut Deppert

**Affiliations:** Solid State Physics and NanoLund, Lund University, Box 118, 22100 Lund, Sweden

**Keywords:** core−shell nanoparticles, molecular dynamics, surface segregation, bimetallic nanoparticles, principal component analysis, linear discriminant analysis

## Abstract

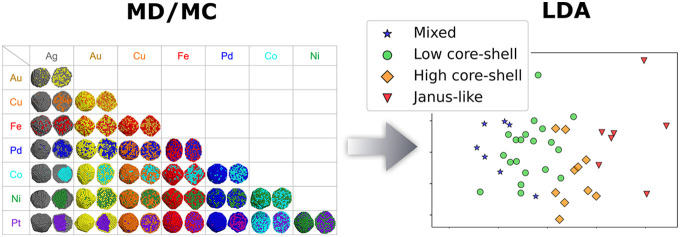

Surface segregation phenomena dictate core–shell preference of bimetallic nanoparticles and thus play a crucial role in the nanoparticle synthesis and applications. Although it is generally agreed that surface segregation depends on the constituent materials’ physical properties, a comprehensive picture of the phenomena on the nanoscale is not yet complete. Here we use a combination of molecular dynamics (MD) and Monte Carlo (MC) simulations on 45 bimetallic combinations to determine the general trend on the core–shell preference and the effects of size and composition. From the extensive studies over sizes and compositions, we find that the surface segregation and degree of the core–shell tendency of the bimetallic combinations depend on the sufficiency or scarcity of the surface-preferring material. Principal component analysis (PCA) and linear discriminant analysis (LDA) on the molecular dynamics simulations results reveal that cohesive energy and Wigner–Seitz radius are the two primary factors that have an “additive” effect on the segregation level and core–shell preference in the bimetallic nanoparticles studied. When the element with the higher cohesive energy also has the larger Wigner–Seitz radius, its core preference decreases, and thus this combination forms less segregated structures than what one would expect from the cohesive energy difference alone. Highly segregated structures (highly segregated core–shell or Janus-like) are expected to form when both the relative cohesive energy difference is greater than ∼20%, and the relative Wigner–Seitz radius difference is greater than ∼4%. Practical guides for predicting core–shell preference and degree of segregation level are presented.

## Introduction

Bimetallic nanoparticles (NPs) with core–shell structures have shown improved performances in magnetics,^1−3^ catalysis,^4−6^ and optics^7,8^ for a variety of applications. They have been successfully created using colloidal synthesis^9,10^ or gas-phase condensation,^11,12^ with flexible control over materials, particle size, and shell thickness. To design core–shell NPs with desired properties, it is crucial to understand the mechanisms that govern core–shell preference when two materials are mixed, that is, whether a core–shell structure will form and, if so, which material occupies the core and which forms the shell. However, predicting core–shell preference is still primarily based on a few experimental observations and limited theoretical studies, and hence the development of new core–shell nanoparticulate materials is normally built on a trial-and-error approach. Here, we present a general trend in core–shell preference for bimetallic NPs that has been found by extensive molecular dynamics (MD) and Monte Carlo (MC) simulation studies carried out for 45 bimetallic NPs.

In theoretical studies, core–shell preference is usually investigated by surface segregation phenomena, which refer to the enrichment of one component of a mixture in the surface region. Surface segregation has been known to depend on the physical properties of the materials, such as the atomic radii, cohesive energy, surface energy, and electronegativity of the core and shell materials.^13^ Density functional theory (DFT) has been used to generate quantitative databases of surface segregation energies. However, the calculated systems are a single impurity in semi-infinite surfaces^14,15^ and in a 55-atom cluster^16^ of metal hosts, and in this sense are not truly bimetallic NPs. On the basis of the database, a general trend of core–shell preference for transient metals was established, placing cohesive energy as the primary factor and Wigner–Seitz radius as the second.^16^ Even though the general trends found by DFT can be used as a guide in designing core–shell NPs, additional calculations are still needed for the specific chemical composition and the nanoparticle size for each specific binary combination. Also, DFT calculation results for a single atom impurity may not be applicable for bimetallic NPs consisting of metals A and B with 0.5(A):0.5(B) composition as the diffusion barrier for biatomic impurities is found to be much higher than for single atoms^17^ and thus core preference changes accordingly.

Global optimization has been employed to find the core–shell preference by calculating the equilibrium structures of bimetallic NPs.^18^ It showed how different equilibrium structures (*e.g.*, mixed, core–shell, Janus) are formed for truncated octahedral nanoalloy NPs depending on the interaction energies. MD is another popular method for studying surface segregation, which has been extensively employed for investigating the segregation phenomenon during the coalescence of two different NPs.^19−25^ Although MD is less accurate than the first-principles calculations based on DFT, it is computationally much less expensive and can thus be a powerful tool for simulating larger NPs. Previous studies so far generally agree on a few rules, *i.e.*, that the more cohesive material occupies the core and the material with the larger atomic radius tends to segregate on the nanoparticle surface on which negative pressure sites are found. However, it is still not clear what the combined effects of different governing factors are. Also, surface energy and cohesive energy are frequently used interchangeably in terms of their effects on the segregation phenomena, which requires clarification.

In this work, we use a combination of MD and MC simulations to predict core–shell preferences for bimetallic NPs and employ the resulting simulation database to outline the general trend to determine the most crucial governing factors and their combined effects on the segregation level of bimetallic NPs. We also elucidate the effect of composition and size of NPs on core–shell preference, which usually are unfeasible to investigate by DFT methods due to computational cost. From the more detailed information on core–shell preference for bimetallic NPs found by MD/MC, we categorize the preferred equilibrium structures into four different types depending on the level of core–shell tendency: mixed, core–shell, highly segregated core–shell, and Janus-like. The categorized data set is subsequently analyzed using principal component analysis (PCA) and linear discriminant analysis (LDA), which have been successfully employed in materials science problems.^26−29^ We use PCA and LDA to determine the deciding factors in core–shell tendency or preferred structures, that is, the surface segregation tendency. On the basis of the simulation database of 45 bimetallic NPs, we provide practical guides for predicting core–shell preference and degree of segregation level.

The general trend observed in the current study can be used as a guide in nanoparticle synthesis methods in which heat-induced surface segregation phenomena play an essential role and in predicting the equilibrium structures of bimetallic NPs. It is also highly relevant for NPs in nonequilibrium states which are extensively studied in catalyst research. High temperature environments are usually required to promote sufficient catalytic activity and morphological transformation of NPs in such an environment leads to a decrease in catalytically active sites. Thus, instability is a major challenge in bimetallic nanoparticle applications. The general trend can be used as a guideline, for example, in creating bimetallic NPs close to the equilibrium structures to increase the stability.

## Results and Discussion

MD/MC simulations were carried out for different particle compositions and sizes. Note that in the following sections where the detailed MD/MC results are discussed, we only present 28 selected combinations. All of the 45 combinations are used in the PCA and LDA analysis in the later section.

### Composition Effect on Core–Shell Preference

#### 0.5(A):0.5(B) Composition

The bimetallic NPs with 0.5:0.5 composition are solidified as the temperature is decreased to room temperature, and their equilibrium structures are found after Monte Carlo simulations are performed. We confirm that each combination forms a crystalline structure by using polyhedral template matching^30^ (Supporting Information (SI), Figure S1). The majority of the combinations form FCC structures, while octahedral BCC structures are observed in some of the bimetallic NPs that contain Fe, for example, Fe–Au.

**Figure 1:**
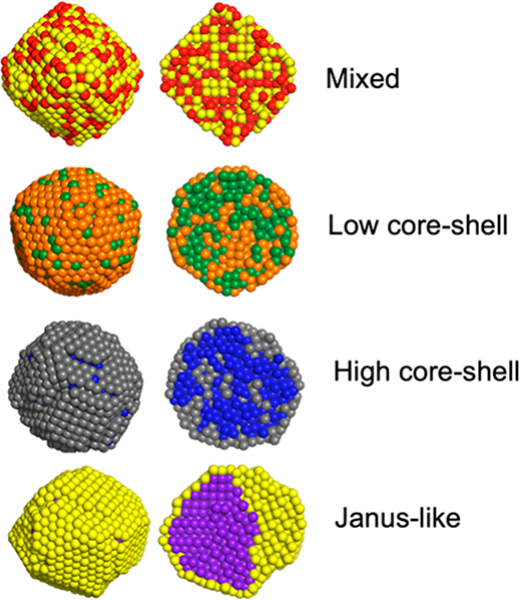
Four different types of bimetallic nanoparticle structures found in the combined molecular dynamics (MD) and Monte Carlo (MC) simulations for 0.5:0.5 composition; mixed, low level of core–shell, high level of core–shell, and Janus-like. The cross-sectional view is placed on the right side.

In crystallized bimetallic NPs, four different types of structures are observed: (a) mixed, (b) low level of core–shell, (c) high level of core–shell, and (d) Janus-like, as illustrated in Figure 1. A mixed structure has a mixture of the elements both in the core and in the shell. A core–shell structure has one material occupying the surface dominantly. A Janus-like structure has a Janus core and a surface composed of a monolayer of one material. We categorized these different types of structures quantitatively using the α-Shapes method.^31,32^ Note that there are other types of bimetallic structures reported for various bimetallic combinations, such as core–satellite,^33^ core–partial shell,^24^ off-center core–shell,^34^ and onion-like.^35^ However, as we are mainly concerned with the surface segregation level in the present study, we categorize structures based on their surface composition. We define the bimetal NPs with one element occupying more than 90% of the surface atom positions as having a high core–shell tendency (high CS). Low core–shell tendency (low CS) is assigned to the bimetallic NPs, with one element occupying more than 70% but less than 90% of the surface atom positions. Mixed is assigned to the combinations with an occupancy ratio of less than 70%. Note that hence, it does not mean that every mixed nanoparticle has a perfect 50:50 mixture on the surface. The run-to-run variability of the simulation results has been checked by carrying out additional simulations on several bimetallic NPs with different random seed numbers. The variations in the results in terms of the surface occupancy ratio are found to be small (0.2% on average).

**Figure 2:**
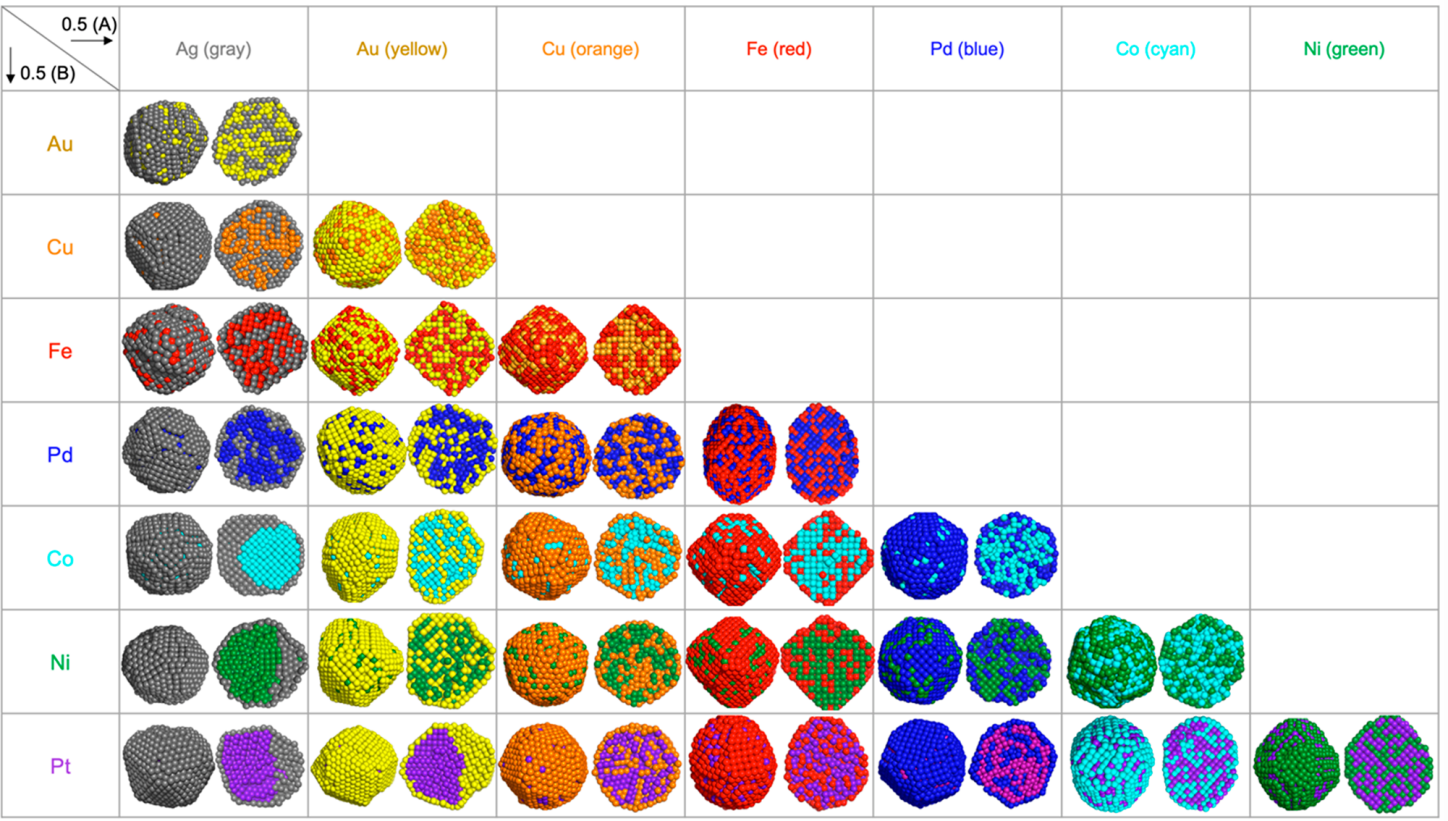
Results of the combined simulations of molecular dynamics (MD) and Monte Carlo (MC) for 0.5(A):0.5(B) composition for 28 combinations of bimetallic nanoparticles.

Figure 2 shows the results of the simulation for the 28 combinations of bimetallic NPs (0.5(A):0.5(B)) showing the preferred structure when they are cooled to room temperature. Mixed structures were found in Cu–Fe, Cu–Pd, Au–Fe, Fe–Pd, and Co–Ni bimetallic nanoparticles. In these mixed structures, neither of the elements shows dominating surface preference. When both materials are liquid at high temperature, the NPs are in mixed structures and remain so even after they are cooled to room temperature and solidified.

Low levels of core–shell structures (low CS) were found in Au–Ag, Fe–Ag, Cu–Au, Pd–Au, Co–Cu, Ni–Cu, Co–Fe, Ni–Fe, Pt–Fe, Ni–Pd, Co–Pd, Pt–Co, and Pt–Ni. High levels of core–shell structures (high CS) were found in Cu–Ag, Pd–Ag, Pt–Cu, Co–Au, Ni–Au, and Pt–Pd. In low and high CS structures, one of the constituent elements clearly exhibits higher surface preference and the other higher core preference. Note that while both materials are in a melted state at high temperature, the surface occupancy ratio is not significantly different from the one after solidification. That is, these low and high core–shell preferences seen after solidification were also observed while both materials are melted.

Janus-like structures were found in Co–Ag, Ni–Ag, Pt–Ag, and Pt–Au. Here, the surfaces are occupied by a single layer of one material, and the core is a distinct Janus structure. The surfaces are formed by a monolayer of Ag in Co–Ag, Ag in Ni–Ag, and Au in Pt–Au, and thus Ag and Au are the surface-preferring materials in these Janus-like structures. Similar to what was observed in the mixed and core–shell structures, even at melted state, the core of the Janus-like structures was not miscible, forming Janus-like structures. Thus, in all types of structures, the segregation level of a certain bimetallic combination in a liquid state is observed to be similar to that of a solid state.

Note that various types of core structures are observed in the mixed and core–shell structures. For example, in Fe–Au and Fe–Pd, the core is somewhat uniformly mixed, whereas the core of Co–Fe, Pt–Pd, and Cu–Ag are relatively segregated. One can also calculate the short-range order (SRO) parameter to determine whether the core is an ordered alloy or a solid solution.^33,36^ As we are more concerned with the surface composition, we leave out further analysis of the core structures.

The occupancy ratios of the atoms in the surface and core in each bimetallic NP after solidification are presented in Table 1 in the column named “0.5(A):0.5(B)”. The core–shell preference found in our MD/MC results generally agree with numerous other segregation studies carried out for various bimetallic NPs such as Cu–Ag,^22,24^ Cu–Au,^37^ Ni–Cu, Co–Au,^38^ Fe–Au,^39^ Ni–Fe, Co–Fe,^40^ Pt–Pd,^41^ and Co–Ag.^42^ There are also disagreements with previous works, for example, the nanothermodynamic approach predicts that Ni segregates on Cu,^43^ and DFT calculations predict surface segregation of Ag on Pt but with a core(Pt)–shell(Ag) structure,^44^ whereas our results predicts Janus-like structure.

**Table 1:** Surface Occupancy Ratios of the Atoms in the Surface and Core in Each Bimetallic NP after Solidification for Different Compositions and Particle Sizes

		composition						size					
		0.5(A):0.5(B)		0.2(A):0.8(B)		0.8(A):0.2(B)		1 nm		6 nm		10 nm	
A	B	surface (A:B)	core (A:B)	surface (A:B)	core (A:B)	surface (A:B)	core (A:B)	surface (A:B)	core (A:B)	surface (A:B)	core (A:B)	surface (A:B)	core (A:B)
Mixed													
Cu	Fe	34:66	57:43	13:87	25:75	70:30	84:16	45:55	64:36				
Cu	Pd	61:39	45:55	27:73	17:83	90:10	74:26	60:40	24:76				
Au	Fe	60:40	45:55	17:83	23:77	95:5	71:29	60:40	24:76	60:40	47:53	64:36	48:52
Fe	Pd	66:34	43:57	25:75	18:82	94:6	71:29	55:45	38:62				
Co	Ni	37:63	56:44	14:86	27:73	71:29	84:16	40:60	76:24				
Low Core–Shell													
Ag	Au	78:22	38:62	38:62	11:89	92:8	73:27	68:32	4:96				
Ag	Fe	77:23	39:61	45:55	8:92	92:8	73:27	66:34	12:88				
Au	Cu	70:30	42:58	27:73	17:83	96:4	71:29	60:40	26:74	71:29	45:55	73:27	47:53
Au	Pd	80:20	37:63	45:55	8:92	98:2	69:31	68:32	4:96	85:15	41:59	85:15	45:55
Cu	Co	86:14	36:64	52:48	6:94	96:4	71:29	69:31	4:96				
Cu	Ni	76:24	40:60	46:54	8:92	92:8	74:26	69:31	0:100				
Fe	Co	80:20	37:63	43:57	9:91	94:6	72:28	70:30	4:96				
Fe	Ni	73:27	40:60	37:63	12:88	91:9	73:27	68:32	7:93				
Fe	Pt	78:22	38:62	19:81	22:78	99:1	69:31	63:37	16:84				
Pd	Ni	71:29	42:58	37:63	13:87	87:13	76:24	67:33	11:89				
Pd	Co	86:14	36:64	47:53	8:92	92:8	73:27	71:29	4:96	83:17	43:57	83:17	45:55
Co	Pt	70:30	41:59	23:77	19:81	95:5	71:29	60:40	32:68				
Ni	Pt	70:30	41:59	25:75	18:82	98:2	70:30	65:35	5:95				
High Core–Shell													
Ag	Cu	98:2	32:68	67:33	1:99	99:1	70:30	74:26	0:100				
Ag	Pd	95:5	31:69	61:39	0:100	99:1	70:30	71:29	0:100	96:4	39:61	96:4	43:57
Cu	Pt	92:8	33:67	36:64	12:88	100:0	69:31	67:33	4:96				
Au	Co	94:6	33:67	54:46	5:95	99:1	69:31	73:27	0:100	93:7	40:60	92:8	44:56
Au	Ni	90:10	34:66	52:48	6:94	99:1	69:31	70:30	0:100				
Pd	Pt	96:4	31:69	58:42	0:100	99:1	69:31	69:31	0:100				
Janus-like													
Ag	Co	100:0	32:68	72:28	0:100	100:0	70:30	75:25	0:100				
Ag	Ni	99:1	33:67	74:26	0:100	99:1	70:30	74:26	0:100	98:2	40:60	99:1	44:56
Ag	Pt	100:0	30:70	62:38	0:100	100:0	69:31	69:31	0:100				
Au	Pt	99:1	29:71	59:41	0:100	100:0	69:31	69:31	0:100	98:2	38:62	98:2	43:57

#### 0.2(A):0.8(B) Composition

MD/MC simulations were carried out for different compositions by varying the number of atoms. In the previous section (0.5(A):0.5(B)), surface- and core-preferring materials were identified in each combination except the combinations that exhibit mixed structures. Here, the number of atoms of the surface-preferring material and the core-preferring material are changed to yield a 0.2 (A): 0.8 (B) composition. In other words, there is less surface-preferring material than core-preferring material. Note that A and B are arbitrarily assigned for mixed structures.

**Figure 3:**
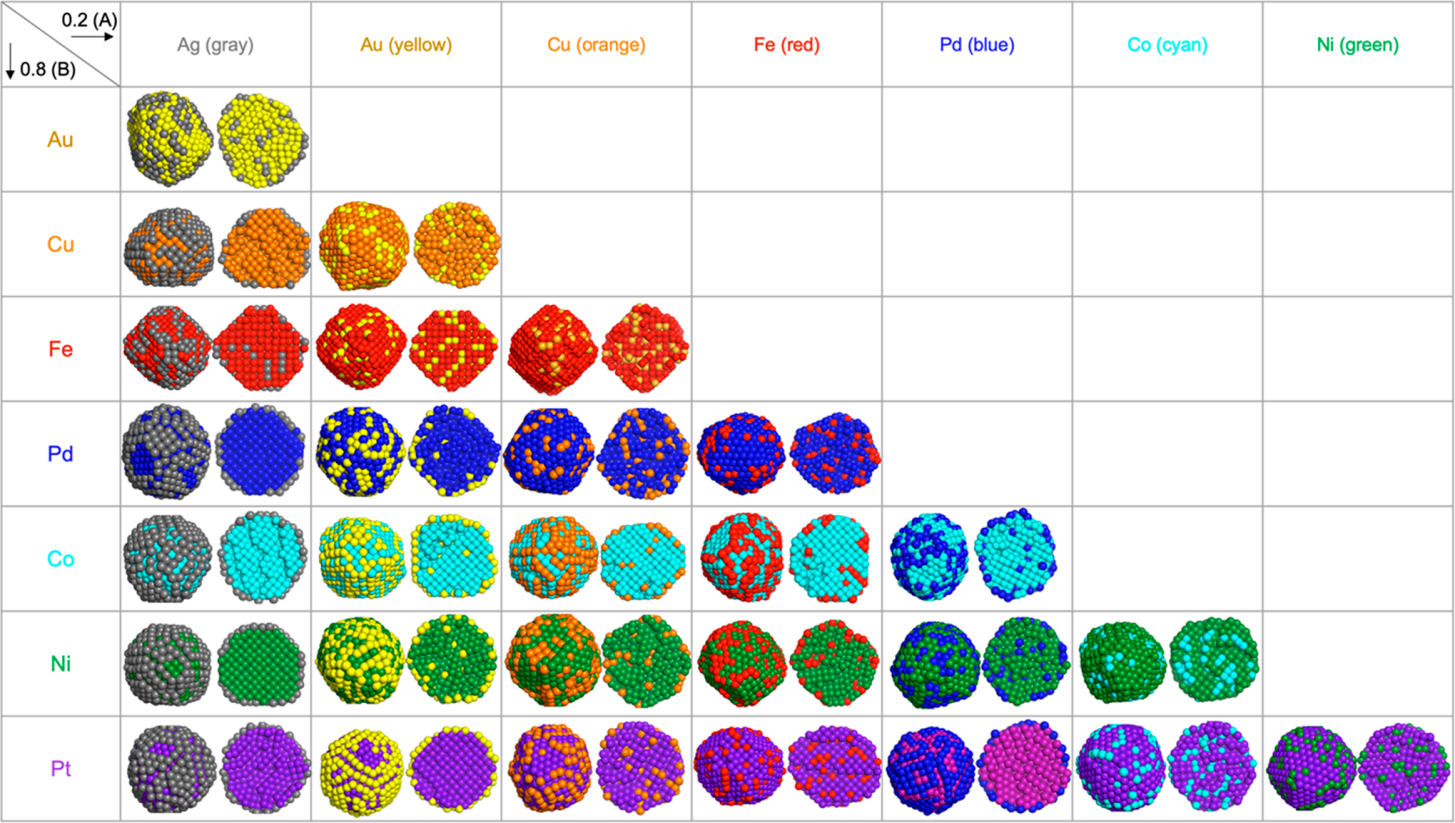
Results of the simulations for the 0.2 (A): 0.8 (B) composition for the 28 combinations of bimetallic nanoparticles. In this composition, there is less surface-preferring material than core-preferring material.

The simulation results for this varied composition are shown in Figure 3, and the surface and core occupancy ratios are listed in Table 1. The combinations identified previously as mixed structures become dominated by the abundant material in both surface and core. In the mixed structures, none of the constituent materials exhibited surface and core preference previously. Hence, when there is an increase in the concentration of one element, the occupancy ratio of that abundant material increases both on the surface and in the core.

In the bimetallic NPs identified as low and high core–shell structures, the surface-preferring material’s surface occupancy decreased, *i.e.*, the surfaces appear patchy. This is seen in the decreased surface occupancy by the surface-preferring material (Table 1). The combinations with high CS preference (Cu–Ag, Pd–Ag, Co–Au, Pt–Pd) also exhibit a patchy shell. However, the core becomes purer with >88% occupancy of the core material. A similar trend is observed in the low CS combinations. Note that some of the patchiness observed in Figure 3 are similar to the “patterned core-partial shell” reported by Grammatikopoulos *et al.*,^24^ in which the spherical Cu core is enclosed by external “cages” of Ag atoms due to the high diffusion rate of Ag on particular Cu facets. We observe similar features in, for example, Pt–Au, Pd–Ag, and Co–Fe in Figure 3. However, we only observed this patchy shell when the surface-preferring material’s composition is low.

The combinations previously identified as Janus-like in the previous section (0.5:0.5 composition) no longer form Janus-like structures in this composition of 0.2(A):0.8(B). Janus-like structures become high core–shell structures with 100% core occupancy by the core-preferring material. This happens because there are not enough surface-preferring atoms left to form one side of the Janus-like structure after preferably forming the surface. The same structural change was reported for Co–Ag bimetallic NPs by Parsina *et al.*(42) Their MD study also showed that the structures of NPs transformed from Janus-like to core–shell depending on the composition.

The patchy surfaces seen in this composition are due to the number of surface-preferring atoms for a given particle size (surface area) not due to the particular composition. As mentioned earlier, the surface-preferring materials tend to preferably form the surface with the excess going into the core. Thus, when there are not enough atoms to form the surface, the surface’s patchiness increases. This means that if the number of atoms of the surface-preferring material increase, then the patchiness will disappear even at the same composition ratio. The effect of increasing the number of surface-preferring atoms was tested with two simulations of larger bimetallic nanoparticles with 0.2(A):0.8(B) composition (SI, Figure S2). The simulation for a 6 nm in diameter NP composed of Ag(0.2):Au(0.8), showed that the patchiness decreased as the Ag surface occupancy ratio improved from 38% to 47%. Similarly, in a 6 nm in diameter bimetallic NP composed of Au(0.2):Pt(0.8), the Au surface occupancy ratio increased from 59% to 97%. In Au–Pt, the surface patchiness disappeared completely. In the later section, increased patchiness is seen in 1 nm NPs for the reason discussed here.

#### 0.8(A):0.2(B) Composition

**Figure 4:**
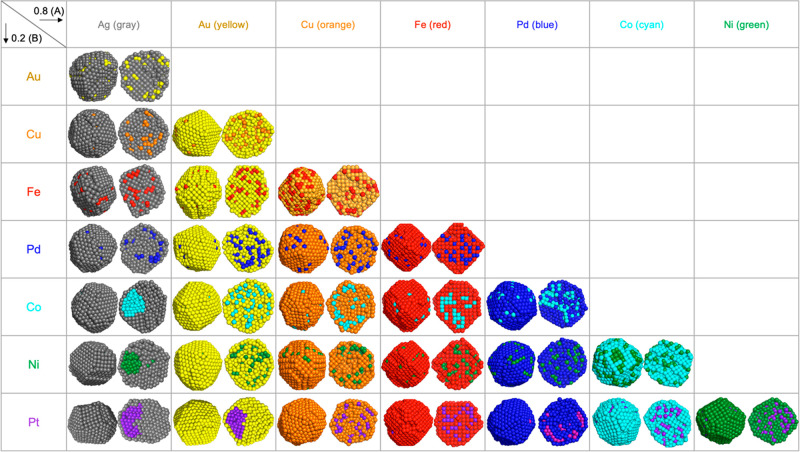
Results of the simulations for a reversed composition: 0.8(A):0.2(B) for the 28 combinations of bimetallic nanoparticles. In this composition, the surface-preferring material is more abundant.

In this part of the work, the composition in each combination is varied oppositely, that is, there is more surface-preferring material (A) than core-preferring material (B). Figure 4 shows the MD/MC results for bimetallic NPs composed of 0.8(A):0.2(B). As in the previous section, the combinations identified as mixed structures become dominated by the abundant material in both surface and core.

The surfaces of the NPs identified as having low and high core–shell tendencies previously are predominantly occupied by the surface-preferring material, which now is the abundant material. In other words, the core–shell tendency has increased in all bimetallic NPs (See Table 1). As the surface-preferring material is abundant even after forming the surface, the core purity inevitably decreases for this composition. The core purity decrease could be interpreted as having a preference for forming a thicker shell by the surface-preferring material. However, we leave this discussion out as we treat and analyze our NPs as having only the outermost atoms belonging to the shell. Janus-like structures have reappeared in Co–Ag, Ni–Ag, Pt–Ag, and Pt–Au. A monolayer of Ag or Au occupies the surfaces. In this composition, there are abundant Ag and Au to form one side of the Janus-like structures after forming the surface.

In all three different compositions studied here, surface- and core-preferring materials in each combination are consistent. However, depending on the sufficiency or scarcity of the surface-preferring material, the degree of core–shell level increases or decreases. The surfaces of the combinations that exhibit low and high CS, and Janus-like structures all become mixed (patchy) when surface-preferring material is insufficient. On the other hand, the trend is reversed, such that even the combinations that display low CS and mixed structures at equal atomic ratio exhibit surfaces predominantly occupied by the abundant material.

### Nanoparticle Size Effect on Core–Shell Preference

Nanoparticle size effects on core–shell preference were investigated by analyzing additional MD/MC simulations carried out for different sizes of bimetallic NPs; 1, 6, and 10 nm in diameter with 0.5(A):0.5(B) composition.

In the smaller bimetallic NPs (1 nm in diameter), the patchiness of the surface is observed in all types of structures (SI, Figure S3). On the other hand, in the core–shell preferring and Janus-like structures, the core purity is increased. The 1 nm in diameter NPs have relatively high surface-to-volume ratio. Thus, most of the surface-preferring atoms occupy the surface and not many of them remain in the core, leading to a high core purity. The relatively low number of surface-preferring atoms explains the commonly observed surface patchiness and increased core purity by the core-preferring material in these small NPs (Table 1).

For the larger NPs with diameters of 6 and 10 nm, eight bimetallic combinations were simulated due to computational cost. Pt–Au and Ni–Ag (Janus-like), Co–Au and Pd–Ag (high CS), Pd–Au, Cu–Au, and Co–Pd (low CS), and Fe–Au (mixed) were chosen as representatives for each structure group (SI, Figure S4). Because of the relatively lower surface-to-volume ratio for the larger particles, the surface occupancy ratios by the surface-preferring materials in these bigger particles are increased compared to the small particles (1 nm) (SI, Figure S5). Note that as the nanoparticle size increases, the purity of the core is decreased in all cases, making the cores more mixed (see Table 1). Similarly, the surface occupancy ratio of Au in the Fe–Au (mixed structure) is increased as well as its core occupancy, making the core more mixed.

This size effects on the segregation observed in our study agree with the predictions based on analytical models,^45,46^ which state that the size effect is due to the decreasing surface-to-volume ratio with increasing particle diameter. The trend found here in different particle sizes is similar to that found in different compositions in that the core–shell preference and the degree of surface segregation of the combinations depend on the sufficiency or scarcity of the surface-preferring material.

### Driving Forces in Core–Shell Preferences

One obvious observation in terms of the different types of structures found in the MD/MC results is that the surface segregation is weakest in the mixed structure and strongest in the high core–shell and Janus-like structures. In the NPs that show Janus-like structures, if the number of atoms of the surface-preferring material is only enough to make the monolayer on the surface, the NPs become perfect core–shell structures. Therefore, it is reasonable to argue that the metals with high segregation tendency form Janus-like structures. We investigated further to find what dictates the trend, and in doing so, to find the driving forces in core–shell preferences.

Principal component analysis (PCA) was performed on the data from the MD/MC results. To have sufficient data points for PCA analysis, 45 data points of MD/MC results for bimetallic NPs (∼4 nm in diameter with 0.5(A):0.5(B) composition), including two additional metals (Al and Mo) were analyzed. The same surface atom identification analysis was performed on the MD/MC results, and each combination was categorized as either mixed, low CS, high CS, or Janus-like. Each data set has eight features, and they are the differences in cohesive energy,^47^ temperature for a given vapor pressure,^48^ atomic radius,^49^ Wigner–Seitz radius,^47,50^ surface energy,^51^ electronegativity,^52^ enthalpy of mixing,^53^ and bulk melting temperature^54^ of the two constituent elements.

**Figure 5:**
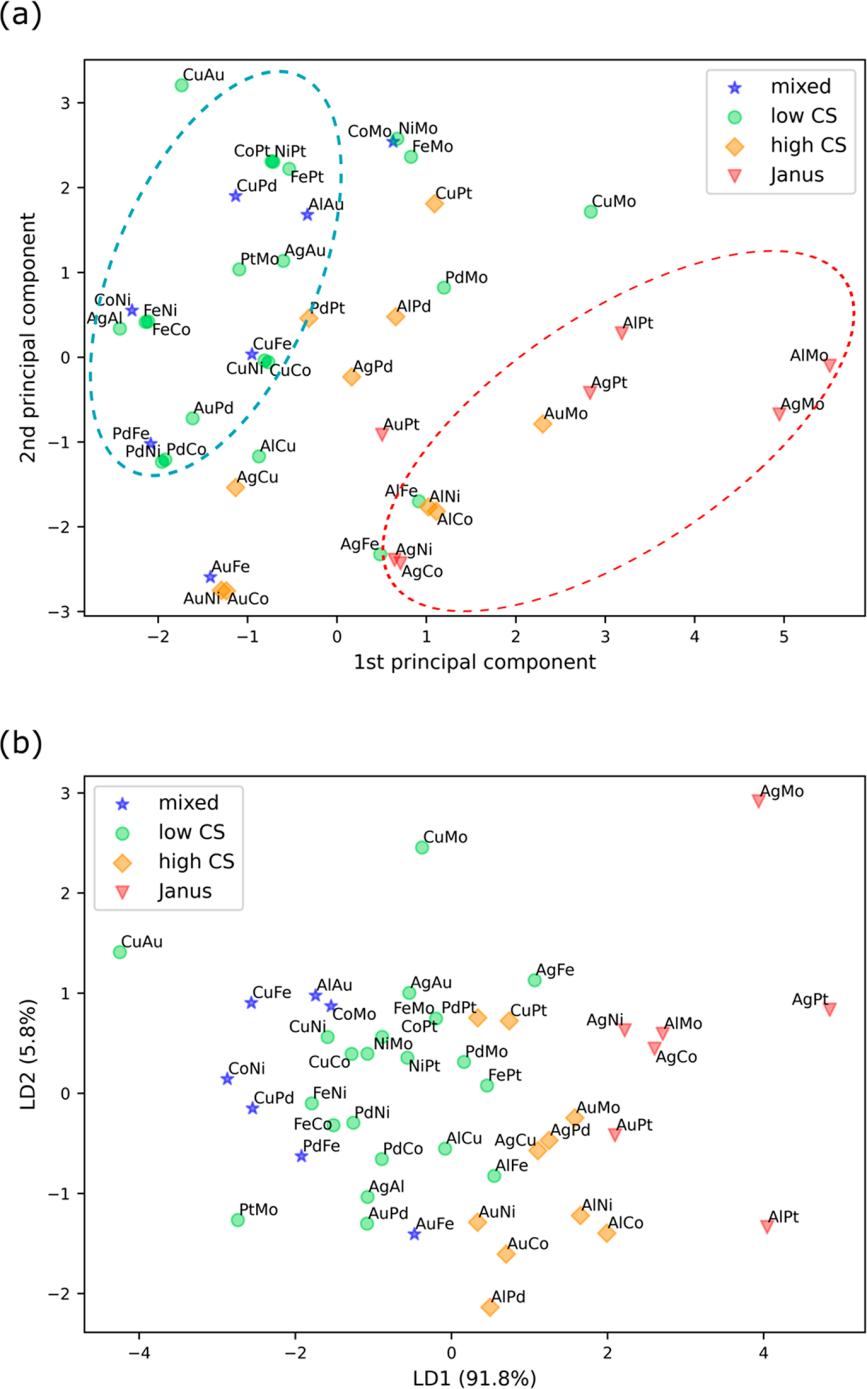
(a) Principal component analysis (PCA) and (b) linear discriminant analysis (LDA) performed on the MD/MC results. In (a), two-component PCA plot for 45 bimetallic nanoparticles used in the analysis shows that Janus-like and mixed structures are well separated from each other. The blue and red dotted line regions are where the low segregation structures and Janus-like structures are mainly positioned, respectively. In (b), the different types of structures are separated by the linear discriminant 1 (LD1) which has high loadings of cohesive energy and Wigner–Seitz radius differences, but has relatively low loadings of surface energy, electronegativity, enthalpy of mixing, and bulk melting temperature differences.

The first three components that reflect 93% of the total variation were considered for PCA analysis after examining the eigenvalues (SI, Figure S6). The PCA score plot of the first two principal components containing 45 data points is shown in Figure 5a. Here, scores are the coordinates of the original data sets in the principal component space. Figure 5a shows that the different types of structures found by MD/MC are reasonably separated by the first and second principal components. The loadings (weights) of each feature in each principal component (SI, Figure S7) revealed that the most prominent factors in the first principal component are the cohesive energy difference (and the difference in the temperature for a given vapor pressure), the surface energy difference, and the bulk melting point difference. Here we will only use cohesive energy without mentioning the vapor pressure temperature as the cohesive energy is a more fundamental concept than the vapor pressure temperature and because the two features are highly correlated. The second principal component’s most prominent factors are the difference in the Wigner–Seitz radius and atomic radius difference. We will only use the Wigner–Seitz radius in the discussion as the two are also highly correlated. The electronegativity and enthalpy of mixing are found to be the prominent factors in the third principal component.

The general trend found from the MD/MC results can be explained by the PCA results in terms of the driving forces. First, the first principal component separates the mixed and Janus-like structures from each other. It shows that bimetallic combinations with high surface segregation, *i.e.*, Janus-like and high core–shell structures, generally appear at the lower right side of the figure (positive side of the first principal component), indicating that they are formed when the cohesive energy (and/or the surface energy and/or the bulk melting temperature) and Wigner–Seitz radius difference are large. On the contrary, mixed structures are generally formed when the cohesive energy and Wigner–Seitz radius difference are small and generally appear on the upper left side of the Figure 5a (negative side of the first principal component).

To get further insight into the PCA results, we performed linear discriminant analysis (LDA) to reduce the data dimension while separating the data based on their class labels (*i.e.*, mixed, low core–shell, high core–shell, and Janus-like). The first two most significant linear discriminants are employed in a two-dimensional plot as shown in Figure 5b. LDA reveals that the different types of structures are separated by the linear discriminant 1 (LD1), which has high loadings of cohesive energy and Wigner–Seitz radius differences, but has relatively small loadings of surface energy, electronegativity, enthalpy of mixing, and bulk melting temperature (SI, Figure S8). Note that the surface energy difference is found to be less effective than cohesive energy in separating the classes and predicting core–shell preference. The LDA supports that the two primary factors are the cohesive energy and the Wigner–Seitz radius differences.

In the majority of the combinations we studied, the element with the higher cohesive energy has the smaller Wigner–Seitz radius. Thus, the general trend is that the element with the higher cohesive energy tends to occupy the core, and cohesive energy is found to be a better predictor for core–shell preference than surface energy or Wigner–Seitz radius. Regarding the degree of segregation, the larger the cohesive energy difference and/or Wigner–Seitz radius difference the higher the segregation level, leading to a high core–shell or Janus-like structure. In fact, Janus-like structures are formed when both the relative cohesive energy and Wigner–Seitz radius differences are large as seen in Figure 5b (see also SI, Figure S9). Molybdenum (Mo) exhibits an unusual property in that it has the highest cohesive energy among the metals studied here but has an unusually large Wigner–Seitz radius. Thus, Mo still occupies the core in most combinations, however, its segregation level is low as seen in, for example, Mo–Au, Mo–Cu, Mo–Ni, and Mo–Co. When the element with the higher cohesive energy has the larger Wigner–Seitz radius, then its core preference decreases, and thus the combination’s surface segregation tendency also decreases. If the cohesive energy difference is not significant, and the Wigner–Seitz radius difference is reversed significantly (that is, the element with higher cohesive energy has a significantly larger Wigner–Seitz radius), the element with the larger cohesive energy can occupy the surface as seen in Cu (core)–Au (shell) and Pt (core)–Mo (shell).

The effect of electronegativity in core–shell preference is controversial. According to the Hume–Rothery rules,^55^ elements with similar electronegativity are likely to form an alloy, but others argue that a large difference in electronegativity favors mixing^56^ due to charge transfer from less to more electronegative elements. Our results agree more with the latter. The combinations with relatively small differences in cohesive energy but significantly large differences in Wigner–Seitz radii, which appear in the lower part of Figure 5a are expected to form high core–shell structures. However, they form low core–shell or mixed structures (for example, Co–Pd, Ni–Pd, Cu–Al, Fe–Au, Fe–Al, Fe–Pd, and Fe–Ag). This might be explained by their relatively large electronegativity differences and charge transfer that favors mixed structures.

On the basis of the MD/MC analysis, we present a guideline for predicting the degree of segregation/mixing in bimetallic combinations. Given that the element with higher cohesive energy has the smaller Wigner–Seitz radius, highly segregated structures (high core–shell or Janus) are formed when both the relative cohesive energy difference is greater than ∼20% and the relative Wigner–Seitz radius difference is greater than ∼4%. However, if the element with the higher cohesive energy has the larger Wigner–Seitz radius, then the combination is likely to form low core–shell or mixed structures. Given that the cohesive energy difference is negligible, such structures with low degrees of segregation may form when the electronegativity difference is large even if the difference in atomic size is significantly large. In short, the cohesive energy dictates the core and surface preference, but the cohesive energy and Wigner–Seitz radius difference together dictate the degree of segregation in an “additive” manner.

**Figure 6:**
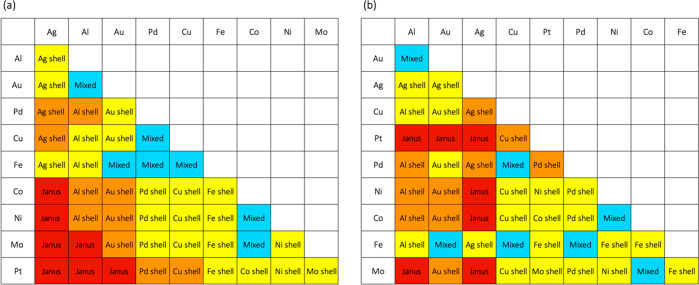
Preferred structures found by MD/MC simulations and LDA for bimetallic nanoparticles. Janus-like (red), high core–shell (orange), low core–shell (yellow), and mixed (blue). (a) Structure preference tabulated in the sequence found by this work using principal component analysis (PCA) and linear discriminant analysis (LDA). (b) Structure preference tabulated in the sequence found by Wang and Johnson.^16^

The MD/MC simulations and the PCA show that materials with high cohesive energy and small Wigner–Seitz radius prefer to occupy the core. The LDA also indicates that cohesive energy and the Wigner–Seitz radius are both critical factors in predicting the preferred structure. On the basis of the LDA results, we scored each element based on their cohesive energy (a higher score for a large cohesive energy) and Wigner–Seitz radius (a higher score for a small radius). The ordering in Figure 6a was calculated using the feature rescaling method (min–max rescaling) and taking the corresponding LDA loadings into account (approximately twice as high a loading for cohesive energy as for Wigner–Seitz radius). The order of the elements in the increasing combined score is Ag < Al < Au < Pd < Cu < Fe < Co < Ni < Mo < Pt. This sequence describes the observed trend well: the metals on the top (or left) prefer to occupy the surface when alloyed with metals on the bottom (or right). Mixed structures are positioned near the diagonal line of the table, and high core–shell and Janus-like structures are positioned toward the left bottom corner.

The sequence found in this study by MD/MC simulations and LDA is somewhat different from the one made by DFT calculations by Wang and Johnson^16^ because their sequence was created first by aligning metals by groups (reflecting the cohesive energy) and then second by aligning them in terms of Wigner–Seitz radius within each group. As shown in Figure 6, weighing the Wigner–Seitz radius, and the cohesive energy by the loadings found by LDA in the ordering scheme is necessary to explain our results, as the sequencing by Wang and Johnson places several highly segregated structures near the diagonal.

Our sequence (Figure 6a) is different from the sequence generated by Wang and Johnson (Figure 6b) in that their sequence was ordered by weighing Wigner–Seitz radius only between the same group of metals in the periodic table. The MD/MC simulation results are not represented well when tabulated in the sequence made by Wang and Johnson (Figure 6b). Note that their sequence is for transition metals only. Moreover, our MD simulations used EAM potentials^57^ for FCC crystals. As shown in the SI, Figure S10, changing to a potential designed for BCC Fe^58^ can alter the structure of the resulting nanoparticle. Thus, one should perhaps not expect to see the same trend as DFT calculations, for example, due to the deviations seen in the combinations that include FCC Fe. The sequence found from the DFT calculation puts Fe as the strongest candidate for the core material in any combinations (Figure 6b). Our simulations do not support this, as Fe exhibits shell-preference in several simulations, but additional simulations using an interatomic potential designed for BCC Fe might support the DFT results. As there are insufficient EAM alloy potentials for bimetallic combinations that include BCC Fe, we focus on FCC potentials here and leave the BCC potentials for future studies.

The general trend found in our study agrees with the effects of cohesive energy and atomic size on bimetallic structures investigated by the global optimization technique.^18^ The study categorized the bimetallic NPs into more different types (*e.g.*, different types of Janus structures and core–shell structures), and thus it may not be easy to directly compare results. However, both results agree in that highly segregated structures (*e.g.*, high core–shell and Janus) are formed between combinations with large cohesive energy difference, and the element with larger atomic size segregates to the surface. We note that there are also disagreements between our results and some of the previous studies in certain aspects. For example, our results contradict the general segregation rules provided by Guisbiers *et al.*,^59^ which were obtained using a nanothermodynamic approach. They found that the primary factor is the bulk melting temperature, and the element with the higher melting temperature segregates to the surface. In our results, melting temperature is found to be not as important as cohesive energy. Also, as melting temperature is loosely, positively correlated with cohesive energy, our results imply that the element with high bulk melting temperature will occupy the core.

When comparing the simulation results of this study to the reported experimental results, it will be ideal to compare with bimetallic NPs in their equilibrium state which can be obtained by incorporating annealing and slow quenching processes during synthesis. We also note that in this study we categorize bimetallic NPs according to the composition of the surface’s outermost layer. If the structure of NPs is determined by techniques such as transmission electron microscopy (TEM), the surface’s outermost layer would not easily be resolved. In that case, one might characterize “low core–shell” NPs as “mixed”. On the other hand, if one focuses only on the characterization of the surface, “Janus-like” NPs could appear as “core–shell”. Thus, experimental results obtained from both surface- and bulk-sensitive characterization techniques will be suitable to be compared with the simulation results presented here. Some experimental reports agree with our simulations in this regard. For example, TEM analysis on Mo–Ni NPs^60^ generated using spark ablation^61^ indicated a mixed structure, but X-ray photoelectron spectroscopy (XPS) on the same system revealed enrichment of Ni on the surface. Tchaplyguine *et al.*(62) investigated Cu–Ag NPs generated by magnetron sputtering, and their results agree with our results on the composition effects on the surface segregation. Using photoelectron spectroscopy, they found that even at similar Cu and Ag fractions, the surface was dominated by Ag (which agrees with Figure 2), and only at low Ag concentration Cu appears on the surface (which agrees with Figure 3). Experimental results on Pt–Pd,^41^ Ni–Co,^63^ and Ni–Au^64^ are also consistent with the simulation results presented here. On the other hand, surface-sensitive analysis on Co–Ag and Ni–Ag^65^ nanoparticles led to the conclusion that the particle morphologies were core–shell whereas our simulations indicate that they form Janus-like structures. There are other experimental results that are seemingly inconsistent with this study.^66,67^ However, we note that the comparisons to the experimental results are not always easy as the surface segregation can be induced by factors other than heat treatment, such as the presence of adsorbates or substrate/support.^68^

## Conclusions

A combination of molecular dynamics (MD) and Monte Carlo (MC) simulations were carried out to predict the core–shell preferences of 45 bimetallic nanoparticle combinations consisting of 10 different metals. Four different structures (mixed, low core–shell, high core–shell, and Janus-like structure) were identified after analyzing the surface atoms of the MD/MC results. The composition and size effect on the preferred structure are investigated. When the surface-preferring material is abundant, the surface is predominantly occupied by the surface-preferring material, and when there are insufficient atoms to form the complete surface layer, the surface becomes patchy or mixed. For the different compositions and particle sizes studied here, we found that the surface segregation and degree of the core–shell tendency depend on the sufficiency or scarcity of the surface-preferring material.

Principal component analysis (PCA) and linear discriminant analysis (LDA) were performed to find the deciding factors and the general trend in the preferred structures and surface segregation tendency. Regarding the prediction of core- and shell-preferring material, cohesive energy is found to be a better predictor than any other investigated factors. However, both Wigner–Seitz radius difference and cohesive energy difference together dictate the degree of segregation in an “additive” manner; the bimetallic combinations with both a significantly large cohesive energy difference (>20%), and a large Wigner–Seitz radius difference (>4%) form highly segregated structures (Janus-like and high core–shell). In the majority of combinations we studied, the element with the higher cohesive energy has the smaller Wigner–Seitz radius, and it occupies the core, which agrees with previous studies. On the other hand, when the element with the higher cohesive energy also has the larger Wigner–Seitz radius, the combination’s surface segregation tendency decreases forming low core–shell structures and in some cases the core–shell preference changes. Even though surface energy is loosely correlated to cohesive energy and thus frequently mentioned as the driving factor in the literature, we found that cohesive energy is a better predictor of core–shell preference. The general trend found in this comprehensive study can be used as a guide when designing bimetallic nanomaterials, especially for gas-phase synthesis methods where the surface segregation phenomenon is an important mechanism.

## Methods

The reliability of MD simulations critically depends on the accuracy of the interatomic potential models. We used the many-body embedded-atom method (EAM)^69,70^ potentials for simulating the interactions between metal atoms in this work. EAM potentials have successfully described properties such as lattice constants, vacancy formation energies, and cohesive energies and are suitable for metallic systems.^19^ They include the effect of local electron density by considering the energy needed to embed a positively charged atom into the electron cloud and the standard pairwise interaction energy between atoms. In the EAM potential, the total energy *E* can be expressed as^70^
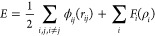

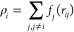
where ϕ_*ij*_ is a pair energy between atoms *i* and *j* separated by a distance *r*_*ij*_, and *F*_*i*_ is the embedding energy term associated with the local electron density ρ_*i*_ calculated from the electron density *f*_*j*_(*r*_*ij*_) at the site *i* arising from atom *j*. Single-element EAM potentials cannot be easily combined to model alloys. Thus, alloy EAM potentials have been constructed from single elemental EAM potentials by normalizing the EAM potentials and introducing an EAM alloy model.^71^ In this work, we use the alloy EAM potentials constructed by Zhou *et al.*,^57^ which provide generalized potentials for any bimetallic combinations of 16 metals. These potentials reproduce basic material properties including sublimation energies and heats of solution. This database of potentials provides a reasonable approximation for the interactions in metallic solutions and has been successfully applied to simulate, *e.g.*, the deposition of multilayer metal systems.^57,72,73^

45 bimetallic NPs composed of 10 metals Ag, Cu, Au, Pd, Fe, Co, Ni, Pt, Al, and Mo are investigated. The starting configuration was a random cloud of 50% A atoms and 50% B atoms (a total of 2600 atoms) placed inside a sphere (4 nm in diameter), the energy of which was quickly minimized using the LAMMPS^74^ energy minimization in a 50 Å × 50 Å × 50 Å box. We denote this first set of simulations as 0.5(A):0.5(B) for the equal composition. MD simulations were conducted using the canonical ensemble conditions (*i.e.*, *NVT*: constant number (*N*), volume (*V*), and temperature (*T*)), and periodic boundary conditions were implemented in all directions. As the simulations started from a melted NP of a mixture of two metals, our study does not consider the effects of nonequilibrium processes, such as coalescence of two nanoparticles and excess thermal energy^75^ during such a collision, which is often studied in microcanonical ensemble (NVE) conditions.^21^ The NPs were initially equilibrated at bulk melting temperature for 40 ps, which was enough for the system to reach equilibrium determined by stabilized potential energy and pressure. The bimetallic NP melts were subsequently cooled at a cooling rate of 0.13 K/ps and equilibrated for 40 ps when the temperature reached 300 K. The Nosé–Hoover thermostat was employed for temperature control. The equations of motion were integrated by the Velocity–Verlet^76^ algorithm with a time step of 1 fs. To ensure that the bimetallic NPs reach the equilibrium state, Monte Carlo was employed after the MD simulation was completed. Monte Carlo has been used to complement MD simulations to find the equilibrium structures of AgCu NPs.^24^ We used the uniform-acceptance force-bias Monte Carlo (fbMC) algorithm^77,78^ implemented in LAMMPS, as it can be easily implemented into an MD simulation code, and it is ideal for solid-state process equilibration. To investigate the effects of the composition, simulations under the same conditions were performed for a bimetallic composition denoted by 0.2(A):0.8(B) and for a reversed composition, 0.8(A):0.2(B). To investigate the particle size effect on the core–shell preference, simulations for 1 nm (90 atoms in total), 6 nm (11 000 atoms in total) and 10 nm (40 000 atoms in total) NPs with 0.5(A):0.5(B) compositions were conducted. All MD simulations were carried out using the LAMMPS^74^ code, and the results were visualized using Pymol^79^ and Ovito.^80^

The degree of core–shell (or surface segregation) tendency was quantified by determining which atoms constitute the surface. Molecular dynamics simulations provide no notion of a surface, except the atomic positions. Therefore, identifying surface and core atoms is done as postprocessing. The identification of surface atoms was carried out based on the Delaunay triangulation of the atomic positions combined with the α-shapes method developed by Edelsbrunner *et al.*(31,32) We chose an α value of 0.6 times the lattice constant of the element with the larger lattice constant, as it yielded a proper identification of the surface atoms in all bimetallic NPs regardless of the different morphologies. Once the surface atoms were identified, depending on the surface occupancy ratio, each bimetallic combination was categorized as one of the four structures: mixed, low level of core–shell, high level of core–shell, and Janus-like. Note that we considered only the outermost layer as the surface (shell) (SI, Figure S11). The effects of cooling rate on the degree of core–shell tendency were investigated by performing MD simulations with different cooling rates (0.013, 0.13, and 1.3 K/ps) for two selected bimetallic combinations, AgPd and CoNi (SI, Figure S12). The results indicate that the segregation level of each combination is consistent throughout the simulations performed at the different cooling rates. We also checked the simulation results for different final temperatures (150, 300, and 450 K) and confirmed that the surface segregation level does not vary significantly (SI, Figure S13). Note that in the Results and Discussion, we show and discuss analysis of the simulation results obtained with a cooling rate of 0.13 K/ps and final temperature of 300 K.

Material properties such as cohesive energy, Wigner–Seitz radius, surface energy, and electronegativity have been suggested as the driving forces in core–shell preference. This study ultimately aims to elucidate the deciding factors in the preferred structures of bimetallic nanoparticles, and in turn, to determine the general trend of core–shell preference. Analyzing multidimensional data such as this is often carried out by principal component analysis (PCA),^81^ a powerful data reduction method. PCA reduces data into a lower-dimensional space spanned by the principal components (PCs) that are linear combinations of original variables (often called features) while retaining the maximum variability (minimum loss of information). Additionally, we compare the PCA results with the linear discriminant analysis (LDA) results. LDA is a similar data dimensionality reduction technique but a supervised approach as it takes the class labels into consideration.^82^

We analyzed the MD/MC results for 45 bimetallic NPs using PCA and LDA to find the relationship between the equilibrium structure (Janus-like, core–shell, mixed) and several physical properties of the materials. Each 45 data set for the bimetallic combinations was assigned to have eight features consisting of differences in cohesive energy,^47^ atomic radius,^49^ Wigner–Seitz radius,^47,50^ experimentally measured surface energy for a liquid metal,^51^ the temperature at which a metal reaches a given vapor pressure (10^–10^ Torr),^48^ electronegativity,^52^ enthalpy of mixing,^53^ and bulk melting temperature^54^ of the core and shell material. Relative difference with respect the smaller of the two values were used as features except for enthalpy of mixing. Feature scaling through standardization, PCA, and LDA calculations were performed using the scikit-learn Python module.^83^

## Supporting Information Available

Figures showing the crystallinity of the bimetallic nanoparticles, the decreased patchiness of the surface, the MD/MC simulation results for 1, 6, and 10 nm in diameter nanoparticles, the surface occupancy ratio of the surface-preferring material in bimetallic nanoparticles as a function of the particle size, the scree and score plots from the PCA, LDA loadings of the first two linear discriminants, MD/MC results plotted in the two-dimensional space spanned only by the relative Wigner–Seitz radius difference and relative cohesive energy difference, the preferred structure for Fe–Cu obtained using MD simulation with interatomic potential for BCC Fe, the identified surface atoms of CuAu system using alpha-shape method, and the effects of cooling rate and final temperature on the degree of core–shell tendency (PDF)
