# Supplementary material for: General Trends in Core–Shell Preferences for Bimetallic Nanoparticles
Source: ACS Nano. 2021 Apr 23;15(5):8883–95. doi: 10.1021/acsnano.1c01500 (PMC8291766; doi:10.1021/acsnano.1c01500)
Supplement: Supplementary file 1 — nn1c01500_si_002.pdf [file nn1c01500_si_002.pdf]

Supporting information

General Trends in Core-Shell Preferences for Bimetallic Nanoparticles

Namsoon Eom, Maria E Messing, Jonas Johansson, and Knut Deppert

Lund University, Solid State Physics and NanoLund, Box 118, 22100 Lund, Sweden

Crystallinity of the bimetallic nanoparticles

The MD simulations were followed by the uniform-acceptance force-bias Monte Carlo (fbMC) so that the bimetallic NPs crystalize. The crystallinity is analysed using polyhedral template matching.

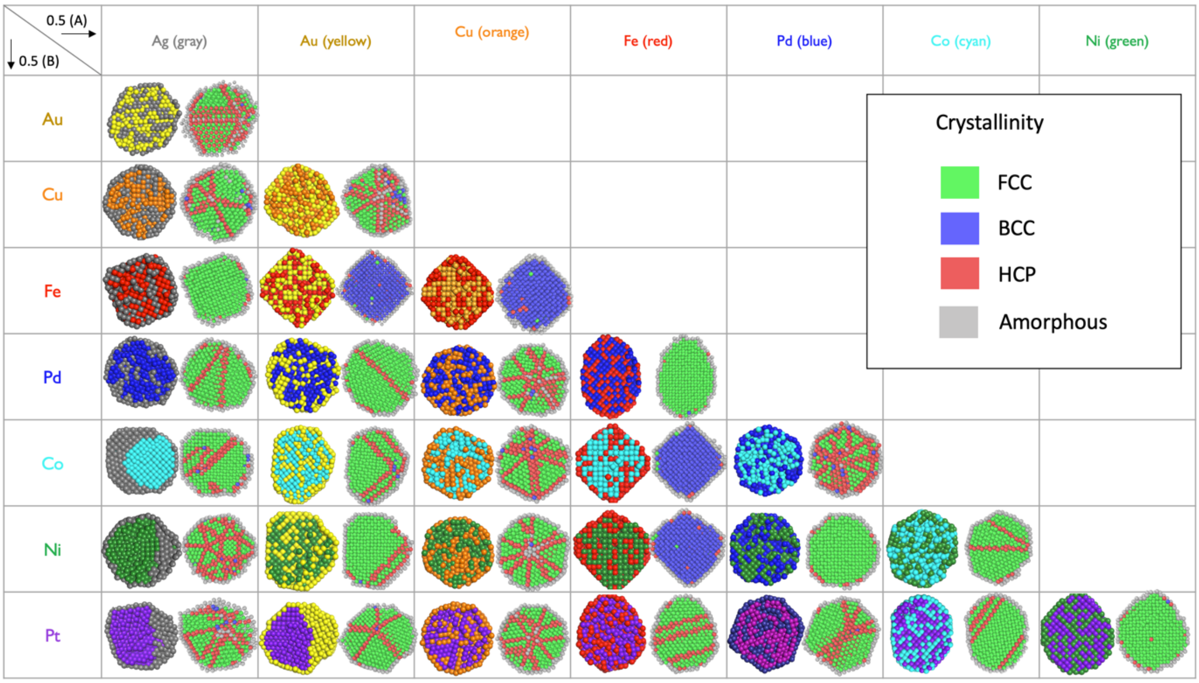

Figure S1. The bimetallic NPs with 0.5 : 0.5 composition after molecular dynamics and Monte Carlo simulations. The crystallinity of each combination is analysed using polyhedral template matching and is shown on the right side. Note that the color scheme for crystallinity only applied to the figures on the right side. The cross-sectional view, where different colors indicate different metal, is shown on the left side.

0.2(A): 0.8(B) composition

The simulation for Ag(0.2)-Au(0.8) 6 nm in diameter nanoparticle, which is in the low core-shell category, showed that the patchiness is decreased. That is, the Ag surface occupancy ratio improved from 38 % to 47 %. Similarly, in Au (0.2) : Pt (0.8) 6 nm in diameter nanoparticle which is categorized as Janus-like, Au surface occupancy ratio improved from 59 % to 97 %. In AuPt, the surface patchiness has disappeared completely. This confirms that the patchy surface seen in the 3 nm bimetallic nanoparticles with 0.2 (A) : 0.8 (B) composition is related to the number of atoms, not to the particular composition.

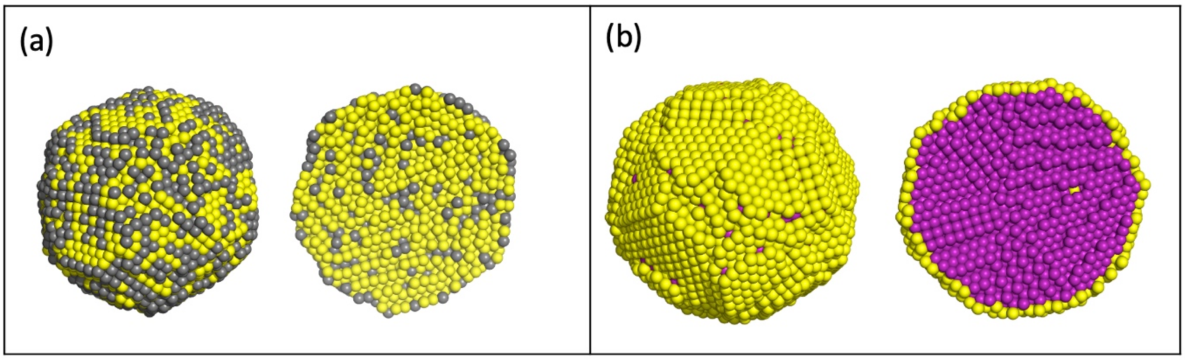

Figure S2. 6 nm in diameter bimetallic nanoparticles composed of 0.2 (A) : 0.8 (B). (a) Ag (grey) and Au (yellow). (b) Au (yellow) and Pt (purple). In both systems, the patchiness of the surface seen in the 4 nm in diameter NPs with the same composition 0.2 (A) : 0.8 (B) in Figure 3 has decreased.

Nanoparticle size effect on core-shell preference

***Small particles**: 1 nm in diameter bimetallic NPs with 0.5 (A) : 0.5 (B) composition*

In the simulations for the small NPs, approximately 73 % of the total number of atoms are surface atoms, on average.

In all types of structures, the patchiness of the surface is observed. Due to a high surface-to-volume ratio for the size of the nanoparticle simulated here, there are relatively few surface-preferring atoms available for forming the surface. Thus, the core purity with the core preferred material is higher in this particle size.

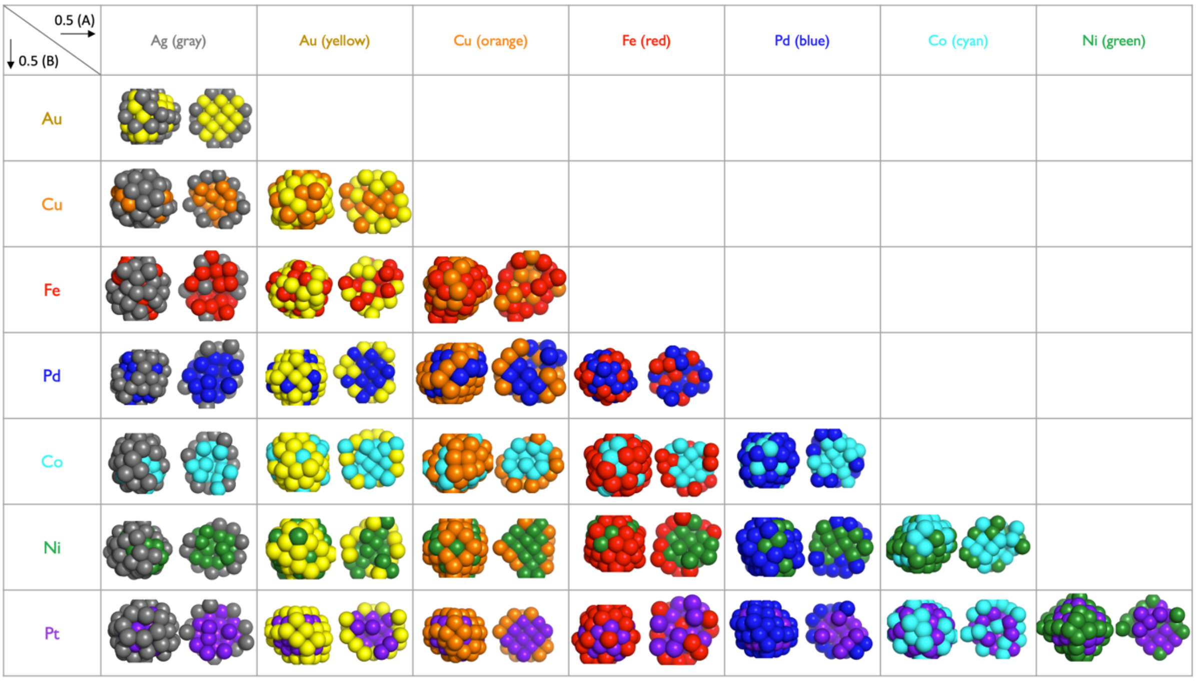

Figure S3. The results of MD/MC simulation for 1 nm in diameter bimetallic NPs for the 28 combinations of bimetallic nanoparticles.

Larger particles:

6 nm and 10 nm in diameters with 0.5 (A) : 0.5 (B) composition

For the larger NPs with diameters of 6 nm and 10 nm, eight bimetallic combinations were simulated due to computational cost. Pt-Au and Ni-Ag (Janus-like), Co-Au and Pd-Ag (high CS), Pd-Au, Cu-Au, and Co-Pd (low CS), and Fe-Au (mixed) were chosen as representatives for each structure group (SI Figure 4).

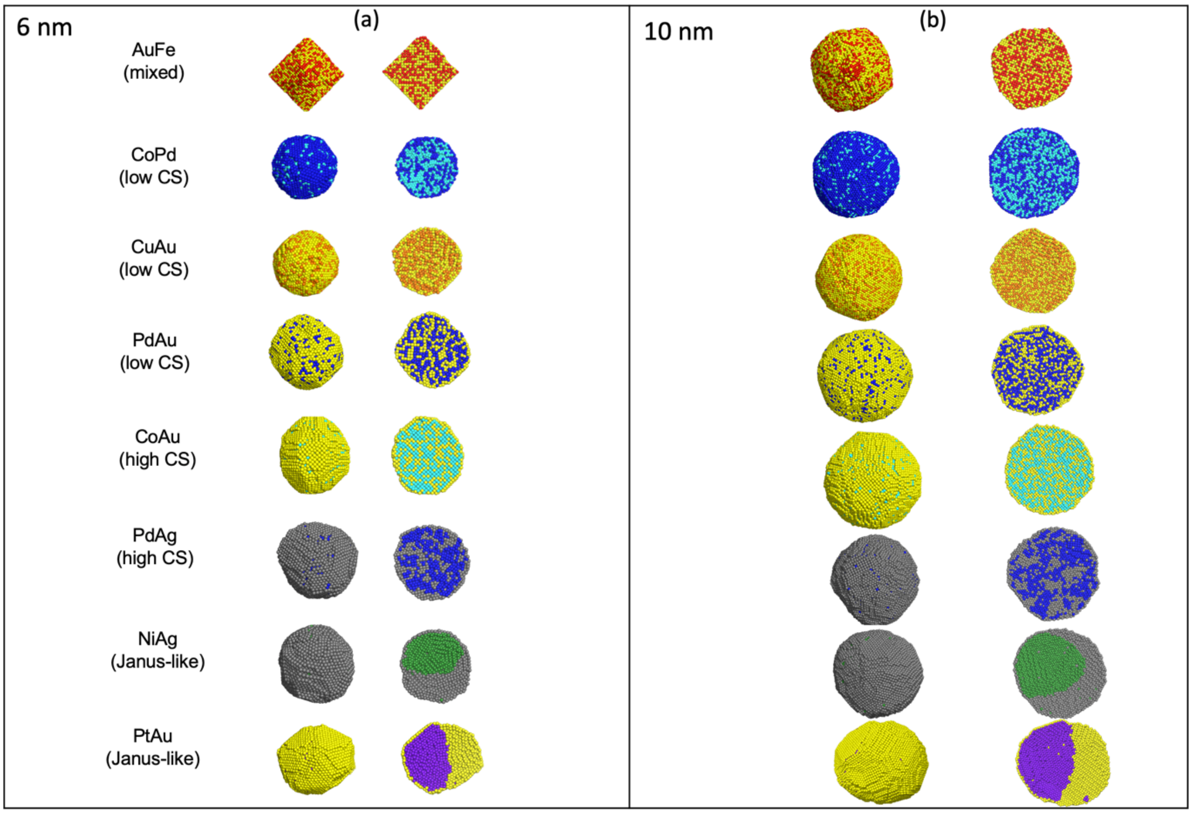

Figure S4. The results of MD/MC simulations for bimetallic nanoparticles; (a) 6 nm in diameter and (b) 10 nm in diameter

Due to the relatively lower surface-to-volume ratio for the larger particles, the surface occupancy ratios by the surface-preferring materials in these bigger particles are improved compared to the small particles (1 nm) (SI figure 5).

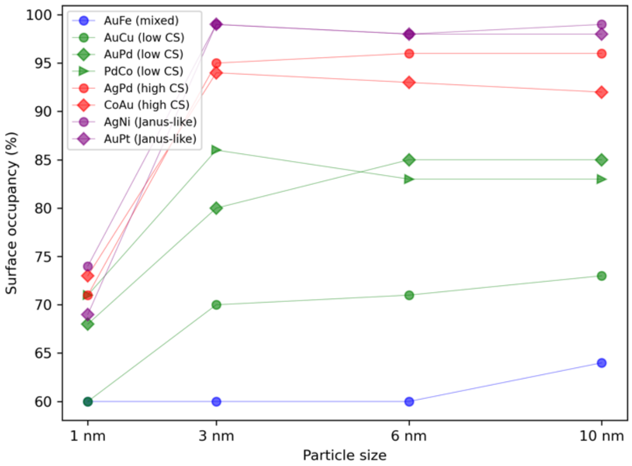

Figure S5. Surface occupancy ratio of the surface-preferring material in bimetallic nanoparticles as a function of the particle size. The surface occupancy ratios by the surface-preferring materials in these bigger particles are increased compared to the small particles (1 nm).

Principal component analysis (PCA)

After running the PCA algorithm on our data, we found that the cumulative variance of the eight-component analysis revealed that the first two principal components and the first three components reflected 74 % and 93 % of the total variation, respectively. For all eight components, we get PC1: 40.8 %, PC2: 33.4 %, PC3: 18.4 %, PC4: 4.2 %, PC5: 2.7 %, PC6: 0.35 %, PC7: 0.17 %, and PC8: 0.03 %. Therefore, we consider the first three principal components in our further analysis.

In PCA, finding the optimal number of principal components (PCs) is done by selecting a set of minimum PCs having a significantly larger Eigenvalue than the rest of PCs. The scree plot shown in SI Figure 6 suggests that the first three principal components adequately explain the variability of the data. Therefore, we used the first 3 principal components whose eigenvalues are greater than approximately 1.

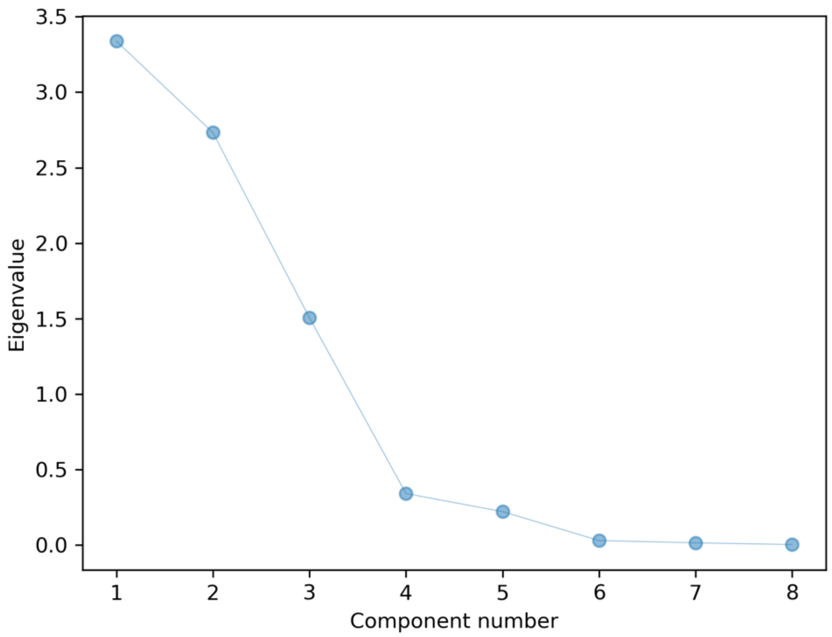

Figure S6. The scree plot of the principal components showing the variance captured.

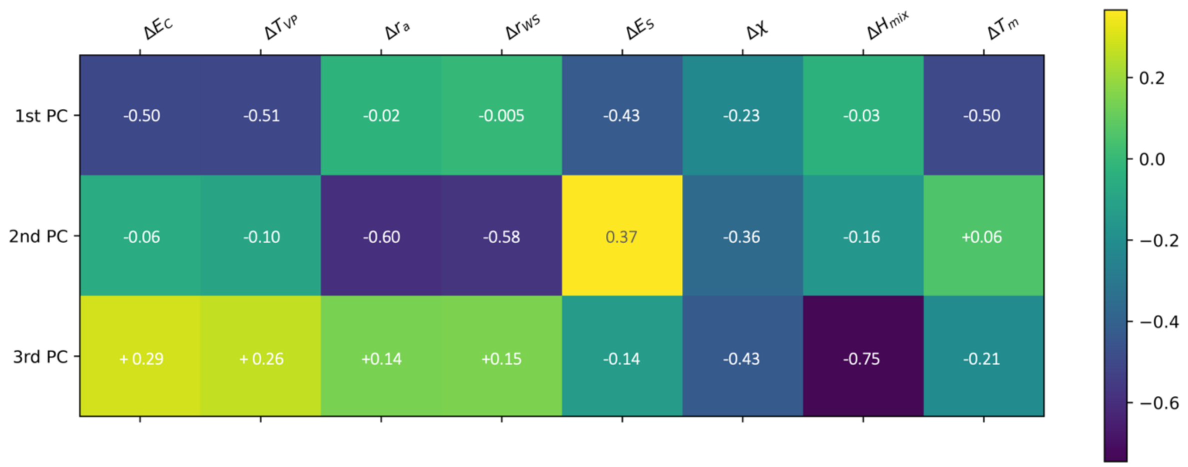

Figure S7. PCA scores of the first two principal components (PCs) for eight features consisting of physical properties which are the differences in cohesive energy (ΔE_C), temperature for given vapor pressure (ΔT_{VP}), atomic radius (Δr_a), Wigner-Seitz radius (Δr_{WS}), surface energy (ΔE_S), electronegativity ($\Delta\chi$), enthalpy of mixing (ΔH_{mix}), and bulk melting temperature (ΔT_m).

Linear discriminant analysis (LDA)

The linear discriminant 1 (LD1) explains 91.8 % of the variance and has high loadings of cohesive energy and Wigner-Seitz radius differences, but has relatively small loadings of surface energy, electronegativity, enthalpy of mixing, and bulk melting temperature. LD2 explains 5.8 % of the variance.

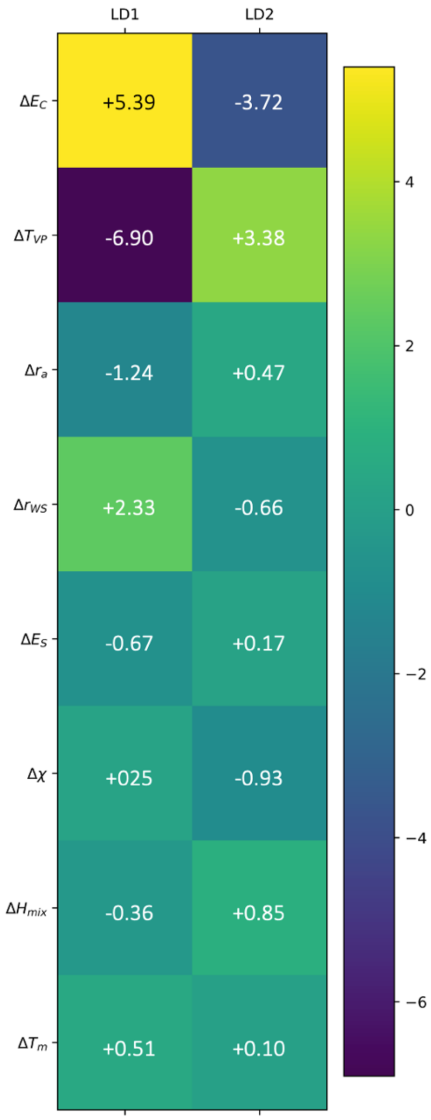

Figure S8. LDA loadings of the first two linear discriminants for eight features consisting of physical properties which are the differences in cohesive energy (ΔE_C), temperature for given vapor pressure (ΔT_{VP}), atomic radius (Δr_a), Wigner-Seitz radius (Δr_{WS}), surface energy (ΔE_S), electronegativity ($\Delta \chi$), enthalpy of mixing (ΔH_{mix}), and bulk melting temperature (ΔT_m).

Relative Wigner-Seitz radius difference *vs* cohesive energy difference

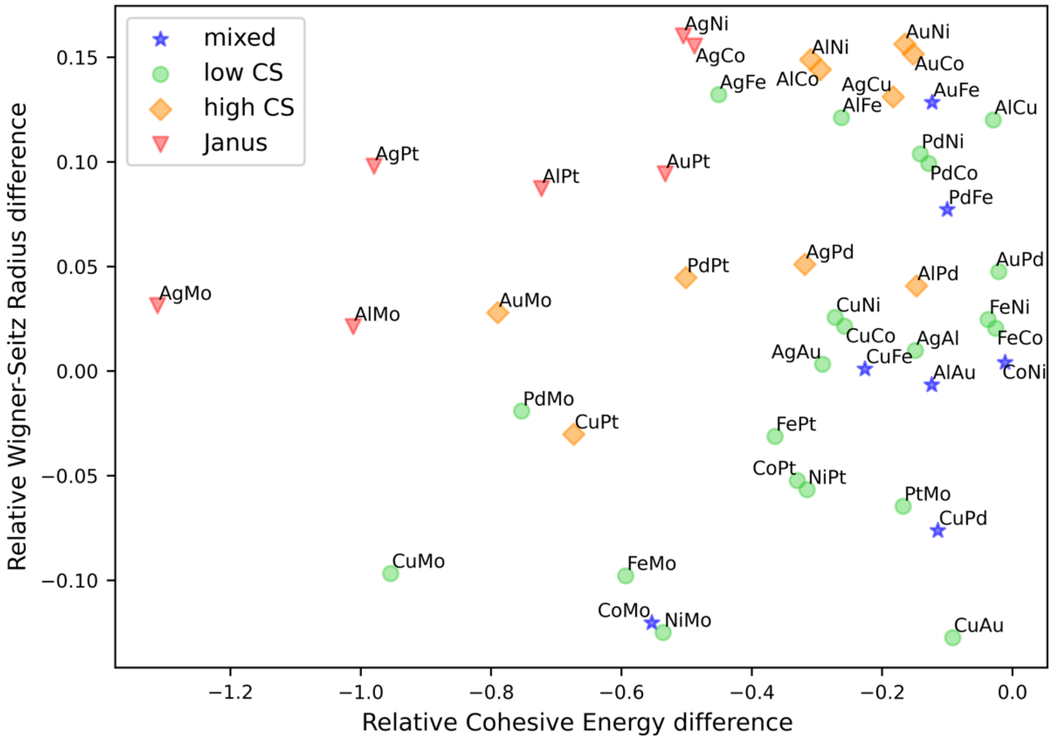

Figure S9. MD/MC results plotted in the two-dimensional space spanned only by the relative Wigner-Seitz radius difference and relative cohesive energy difference

A simulation using a BCC Fe interatomic potential

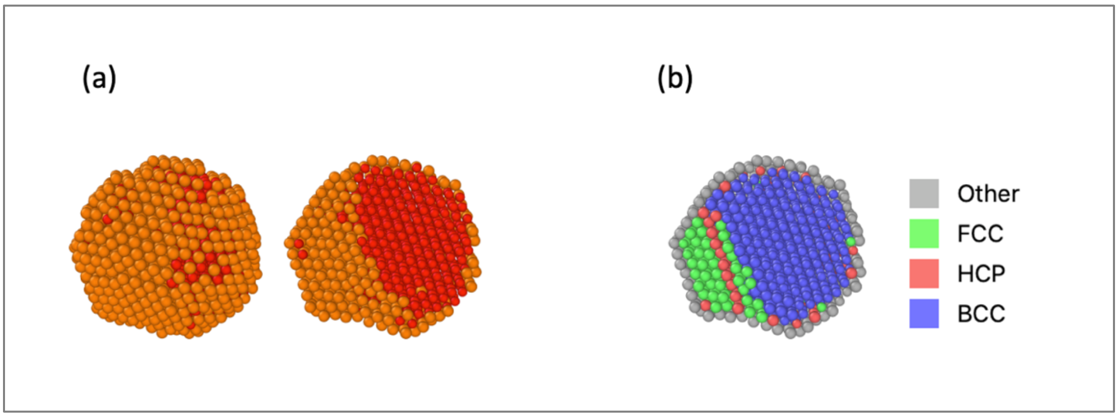

Figure S10. The preferred structure for Fe-Cu was obtained using MD simulation with interatomic potential for BCC Fe. (a) full view(left) and cross-sectional view (right) of the bimetallic NP with strongly core-preferring Fe (red) and surface-preferring Cu(orange). The structure is a highly segregated Janus-like. (b) The crystalline structure of the Janus-like Fe-Cu bimetallic NP.

Surface atom identification using alpha-shape

Using the alpha-shape method, the surface atoms of each system are identified. SI Figure 11 shows an example of the results. Here the CuAu system is analysed using an alpha value of 2.45 (*i.e.,* 0.6 times the lattice constant of Au), which enable the surface atoms to be distinguished from the core atoms.

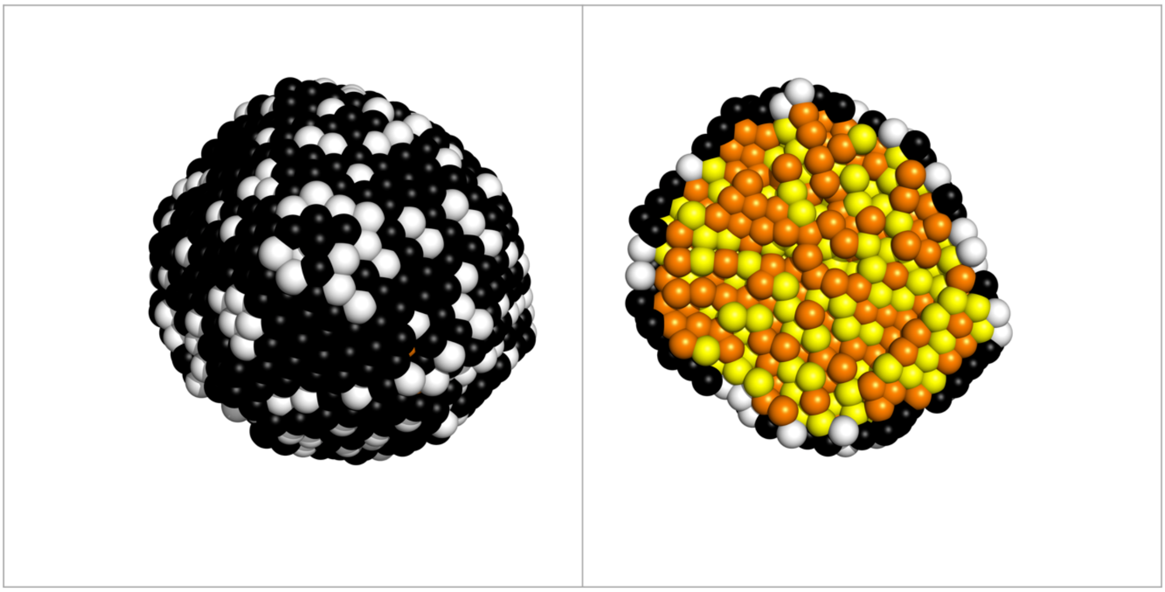

Figure S11. Identified surface atoms of CuAu system. Atoms are color coded: Surface Cu atoms: white, surface Au atoms: black, Cu core atoms: orange, Au core atoms: yellow.

Effects of cooling rate on the surface segregation

Effects of cooling rate on the degree of core-shell tendency were investigated by performing MD simulations at different cooling rates (0.013 K/ps and 1.3 K/ps). The simulations were carried out for AgPd (high core-shell) and CoNi (mixed). The results indicate that the segregation level is consistent throughout the simulations performed at the different cooling rates.

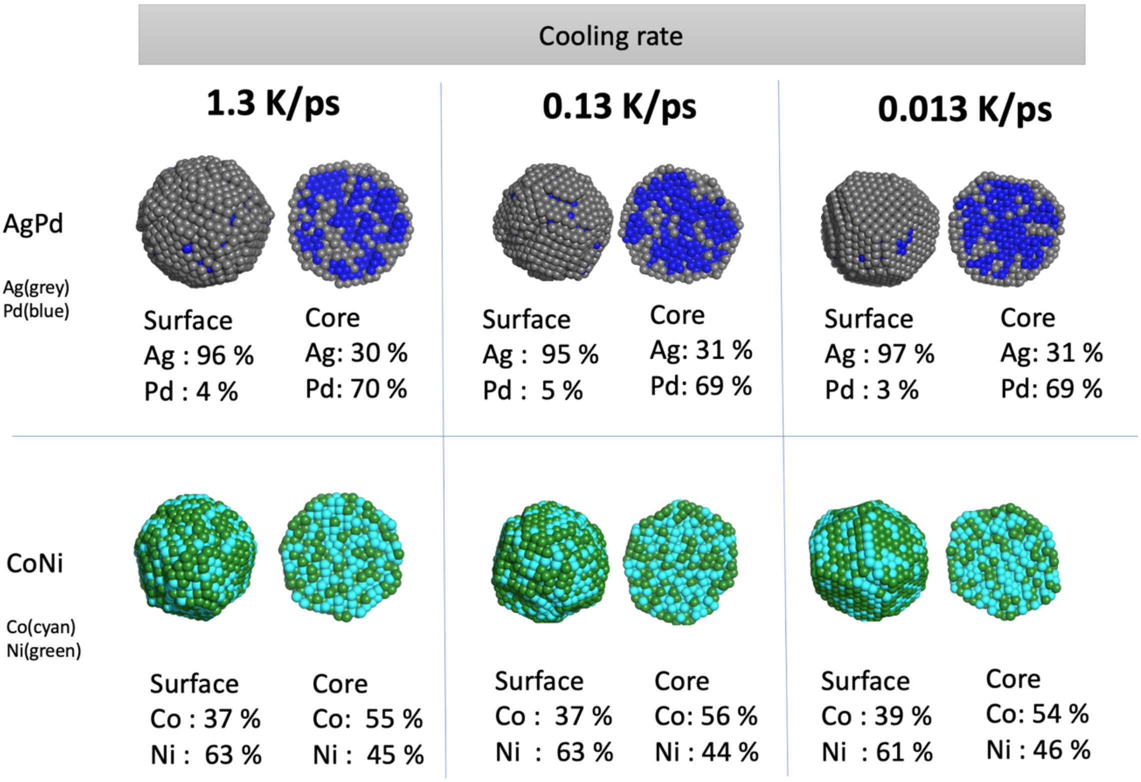

Figure S12. Effects of cooling rate on the degree of core-shell tendency for AgPd and CoNi. In AgPd, Ag is the surface-preferring material with a surface occupancy ratio approximately 95 %. In CoNi, the surface composition is mixed as the occupancy ratio of neither of the elements is above 70 %.

Effects of final temperature

Effects of the final temperature on the degree of core-shell tendency were investigated by performing the simulations with different final temperatures: 150 K, 300 K, and 450 K for AgPd and CoNi. Note that the cooling rate was set to 0.13 K/ps for all simulations. The segregation level is found to be consistent with negligible fluctuations in the surface and core occupancy ratios.

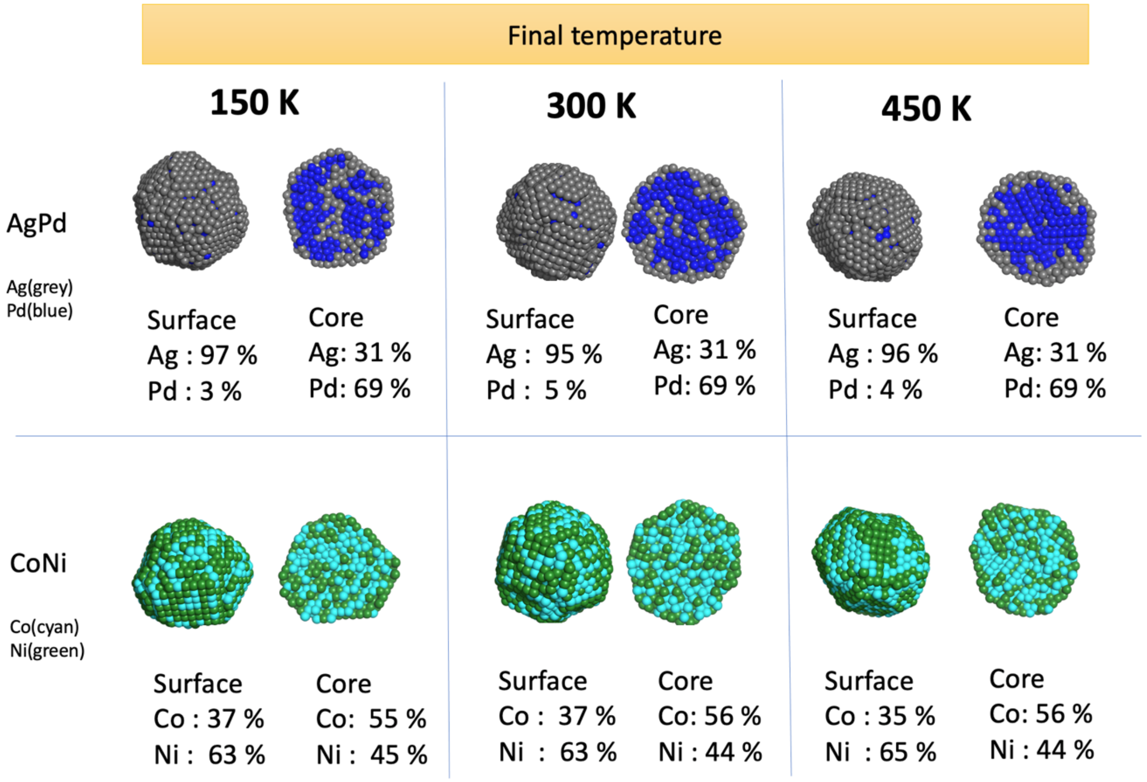

Figure S13. Surface and core occupancy ratio of each element of the simulations obtained with different final temperatures.
